# Blood lactate is a predictor of short-term mortality in patients with myocardial infarction complicated by heart failure but without cardiogenic shock

**DOI:** 10.1186/s12872-018-0744-1

**Published:** 2018-01-18

**Authors:** Grunde Gjesdal, Oscar Ö. Braun, J. Gustav Smith, Fredrik Scherstén, Patrik Tydén

**Affiliations:** Department of Cardiology, Clinical Sciences, Lund University and Skåne University Hospital, SE-221 81 Lund, Sweden

**Keywords:** Lactate, Acute coronary syndrome, Myocardial infarction, Killip class, Cardiogenic shock

## Abstract

**Background:**

Mortality in patients with acute myocardial infarction (AMI) has improved substantially with modern therapy including percutaneous coronary interventions (PCI) but remains high in certain subgroups such as patients presenting with overt cardiogenic shock. However, the risk for AMI in patients presenting acutely with signs of heart failure but without cardiogenic shock is less well described. We aimed to identify risk factors for mortality in AMI patients with heart failure without overt cardiogenic shock.

**Methods:**

Using data from the Swedish Coronary Angiography and Angioplasty Registry (SCAAR), we identified patients with operator-registered heart failure (Killip class II-IV), and evaluated predictors of mortality based on clinical factors from review of patient records.

**Results:**

A total of 1260 unique patients with acute myocardial infarction underwent PCI in 2014, of which 77 patients (7%) showed signs of heart failure (Killip II-IV) Overall 30-day mortality in patients with Killip class II-IV was 20% (*N* = 15). In patients classified Killip IV (1%), 30-day mortality was 50% (*N* = 6). In patients presenting with mild to moderate heart failure (Killlip class II-III), 30-day mortality was 14% (*N* = 9). In patients with Killip class II-III, lactate ≥2.5 mmol/L was associated with 30-day mortality, whereas systolic blood pressure < 90 mmHg, age, sex and BMI were not. In patients with lactate < 2.5 mmol/L 30-day mortality was 5% (*N* = 2) whereas mortality was 28% (*N* = 7) with lactate ≥2.5 mmol/L. This cut-off provided discriminative information on 30-day mortality (area under ROC curve 0.74).

**Conclusions:**

In patients with AMI and signs of mild to moderate heart failure, lactate ≥2.5 mmol/L provides additional prognostic information. Interventions to reduce risk may be targeted to these patients.

## Background

Since Thomas Killip and John T Kimball proposed a classification for heart failure in patients with acute myocardial infarction (AMI) [1] (Table 1), mortality in AMI has improved significantly [1, 2]. Although prognosis for stable patients has improved, mortality remains high in patients with impaired hemodynamics and especially in those with cardiogenic shock [3–5]. For these patients, the only treatment proven to improve mortality is early revascularization [6, 7] while use of mechanical support devices such as intra-aortic balloon pump counterpulsation and left ventricular assist devices such as the Impella pump™ has not been proven to reduce mortality [8, 9]

**Table 1:** Killip classification

Killip class I:	Individuals with no clinical signs of heart failure.
Killip class II:	Individuals with rales or crackles in the lungs, an S3, and elevated jugular venous pressure.
Killip class III:	Individuals with frank acute pulmonary edema.
Killip class IV:	Individuals in cardiogenic shock or hypotension (measured as systolic blood pressure lower than 90 mmHg) and evidence of peripheral vasoconstriction (oliguria, cyanosis or sweating).

.

While patients presenting with cardiogenic shock in the context of AMI have been thoroughly investigated [4, 5, 10], less attention has been paid to prognostic markers of short term mortality in patients with AMI and signs of heart failure but without overt cardiogenic shock. Factors such as age, previous myocardial infarction, cardiothoracic ratio on chest X-ray, blood urea and lactate concentrations have all been shown to influence mortality in patients with AMI [11], but there may still be subgroups within these apparently stable patients with mortality rates worthy of extra attention.

Blood lactate is an established prognostic marker in septic shock [12, 13] and in intensive care unit patients in general [14, 15]. In septic patients with stable hemodynamics, elevated lactate concentrations have been associated with future need for vasopressor support [16]. In patients presenting with AMI and cardiogenic shock, several studies have shown the usefulness of lactate as a prognostic marker [17, 18]. Furthermore, elevated blood lactate at presentation has even been associated with increased mortality in patients with acute decompensated heart failure in general [19]. In 1991 Mavric et al [11] showed a relationship between elevated blood lactate and later development of shock even in patients with Killip classes I and II, but such a relationship has not yet been proven after the introduction of percutaneous coronary intervention (PCI).

In this study, our aim was to identify whether blood lactate concentration could be used as a prognostic marker in patients presenting with AMI with signs of heart failure but without pronounced hemodynamic impact and absence of hypotension.

## Methods

### Study design

Using data from the Swedish Coronary Angiography and Angioplasty Registry (SCAAR), we identified patients treated for primary AMI at Skåne University Hospital in Lund during the period of January 1st to December 31st 2014. For patients who underwent more than one angiography during the time-period, the analysis was restricted to the first occasion. Prior to data extraction, the institutional review board exempted the study from formal review.

### Inclusion criteria and data collection

On admission to the catheter lab, patients had blood drawn for various analyses including lactate, according to clinical routine, and were assigned to Killip classes I-IV according to absence or grade of heart failure. Patients presenting with some degree of operator-classified heart failure (Killip class II-IV) were selected. Patients classified in Killip class I, i.e. no signs of no heart failure, were excluded from the analysis,.

In all patients in Killip class II and above, data on age, sex, BMI and mortality at 30 and 365 days were collected from the SCAAR registry. Systolic blood pressure and blood lactate on arrival was collected from the electronic medical records.

### Statistical analysis

Normal distribution was confirmed by visual inspection of histograms. Results are presented as numbers (N) and percentages (%). Difference in mortality was assessed by Kaplan-Meier survival curves and the log rank test. Cox regression analyses were performed to assess statistical significance of lactate and known variables affecting survival (hypotension, age, sex, BMI), and clinical significance was demonstrated by ROC-analysis. A threshold value for elevated blood lactate was set at > 2.5 mmol/L for the statistical analysis. All statistical analyses were performed in SPSS version 24 (IBM Corporation, Armonk, NY, USA).

## Results

**Fig. 1:**
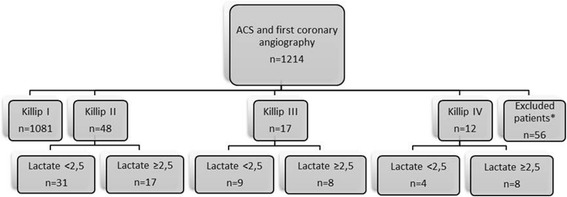
Patient flow-chart showing patient population and selection. ACS = acute coronary syndrome

A total of 1260 patients with acute primary MI underwent PCI in Lund 2014. Patients with ST-segment elevation complicated by out of hospital cardiac arrest (*N* = 27), and patients with AMI seeking care more than 24 h after symptom debut (*N* = 28) and rescue PCI (*N* = 1) were excluded from the analysis on the basis of well-established high baseline mortality. For 46 patients, Killip classification was not available and these patients were excluded from further analysis (Fig. 1). Baseline characteristics are presented in Table 2. Angiographic findings for patients with Killip II-III are presented in Table 3.

**Table 2:** Baseline patient characteristics

	Killip I (*n* = 1081)	Killip II (*N* = 48)	Killip III (*N* = 17)	Killip IV (*N* = 12)	Total (*N* = 1158)
BMI (mean/range) *p* = 0.29	27 (12–51)	27 (15–37)	25 (20–36)	27 (22–31)	27 (12–51)
Age (mean/range) *p* = 0.49	67 (28–95)	72 (47–93)	73 (45–86)	67 (45–82)	67 (28–95)
Male sex *p* = 0.15	698 (65%)	32 (67%)	11 (65%)	9 (75%)	750 (65%)
Smoker *p* = 0.79	314 (29%)	7 (15%)	3 (18%)	4 (33%)	328 (28%)
Former smoker *p* = 0.66	412 (38%)	15 (31%)	6 (35%)	4 (33%)	437 (38%)
Diabetes Mellitus *p* = 0.74	207 (19%)	16 (33%)	8 (47%)	3 (25%)	234 (20%)
Hypertension *p* = 0.31	565 (52%)	27 (56%)	12 (71%)	8 (67%)	612 (53%)
Hyperlipidaemia *p* = 0.37	352 (33%)	14 (29%)	7 (41%)	3 (25%)	376 (33%)
Prior ACS *p* = 0.10	209 (19%)	11 (23%)	3 (18%)	2 (17%)	225 (19%)
Prior PCI *p* = 0.33	168 (16%)	7 (15%)	1 (6%)	0	176 (15%)
Prior CABG *p* = 0.25	62 (6%)	5 (10%)	2 (12%)	0	69 (6%)
Creatinine clearance *p* = 0.66	87	72	78	62	86

For BMI and age data are presented as mean and range within brackets

For Creatinine clearance data are presented as mean

*P*-value from Cox regression analysis regarding the parameters relation to 30-day mortality stratified by Killip group

*ACS* acute coronary syndrome, *BMI* body mass index, *CABG* coronary artery bypass graft, *PCI* percutaneous coronary intervention

**Table 3:** Angiographic finds in patients Killip class II-III

	Frequency	Percentage
1-VD without mainstem	11	17
2-VD without mainstem	13	20
3-VD without mainstem	15	23
Main stem stenosis	21	32
Normal/atheromatosis	5	8
Total	65	100

*VD* vessel disease

We identified 1081 (93%) patients classified in the catheterization laboratory as being free from signs of heart failure (Killip class I). 30-day mortality in this group was 3% (*N* = 32) and 1-year mortality was 8% (*N* = 85).

**Fig. 2:**
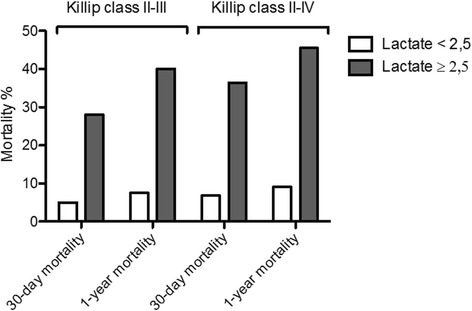
30-day and 1-year mortality in patients with AMI and Killip class II-III presenting with normal (< 2.5 mmol/L) compared to elevated (≥2.5 mmol/L) blood lactate

Seventy-seven patients (7%) showed signs of heart failure (Killip II-IV), and had an overall mortality at 30 days and 1 year of 20% (*N* = 15) and 25% (*N* = 19) respectively (Fig. 2).

Patients who had signs of heart failure, but were not in shock and did not have symptomatic hypotension (Killip class II-III) had an overall 30-day mortality of 14% (*N* = 9). 1-year mortality in this group was 20% (*N* = 13) (Fig. 2). Two patients registered as Killip class II or III presented with systolic blood pressure below 90 mmHg. Both were alive after 1 year.

In patients with cardiogenic chock at presentation, defined as Killip class IV (1%), mortality was 50% (*N* = 6) both after 30 days and 1 year.

**Fig. 3:**
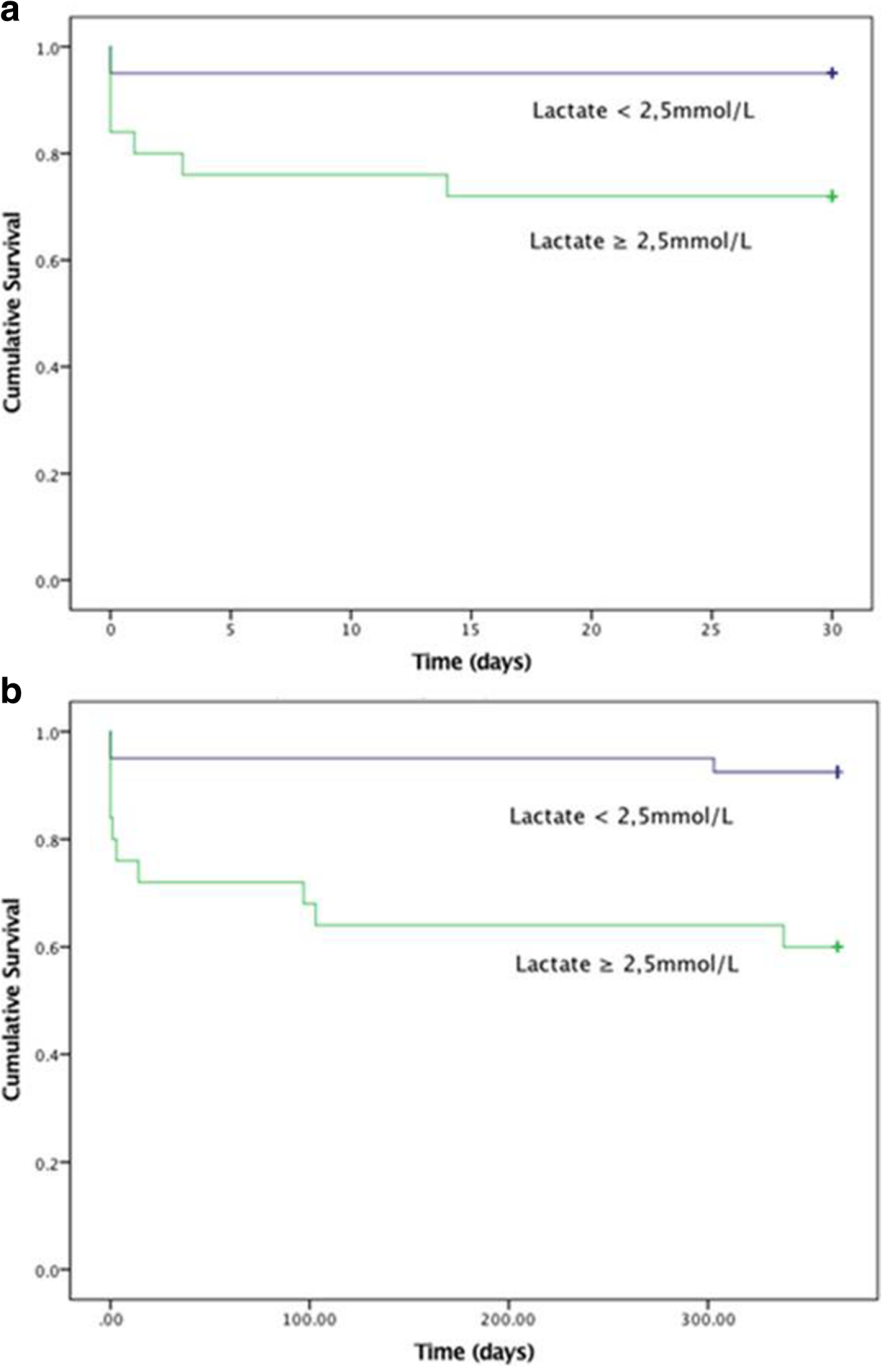
**a** Kaplan-Meier curve showing 30-day mortality in patients presenting in Killip class II-III with and without elevated lactate defined as blood lactate ≥2.5 mmol/L. **b** Kaplan-Meier curve: 1-year mortality in patients presenting in Killip class II-III with and without elevated lactate defined as blood lactate ≥2.5 mmol/L

In patients with Killip class II-IV, but blood lactate < 2.5 mmol/L, 30-day mortality was 7% (*N* = 3) compared to 36% (*N* = 12) in patients presenting with blood lactate ≥2.5 mmol/L. Mortality in the same groups at 1 year were 9% (*N* = 4) and 46% (*N* = 15) respectively (Fig. 3).

**Fig. 4:**
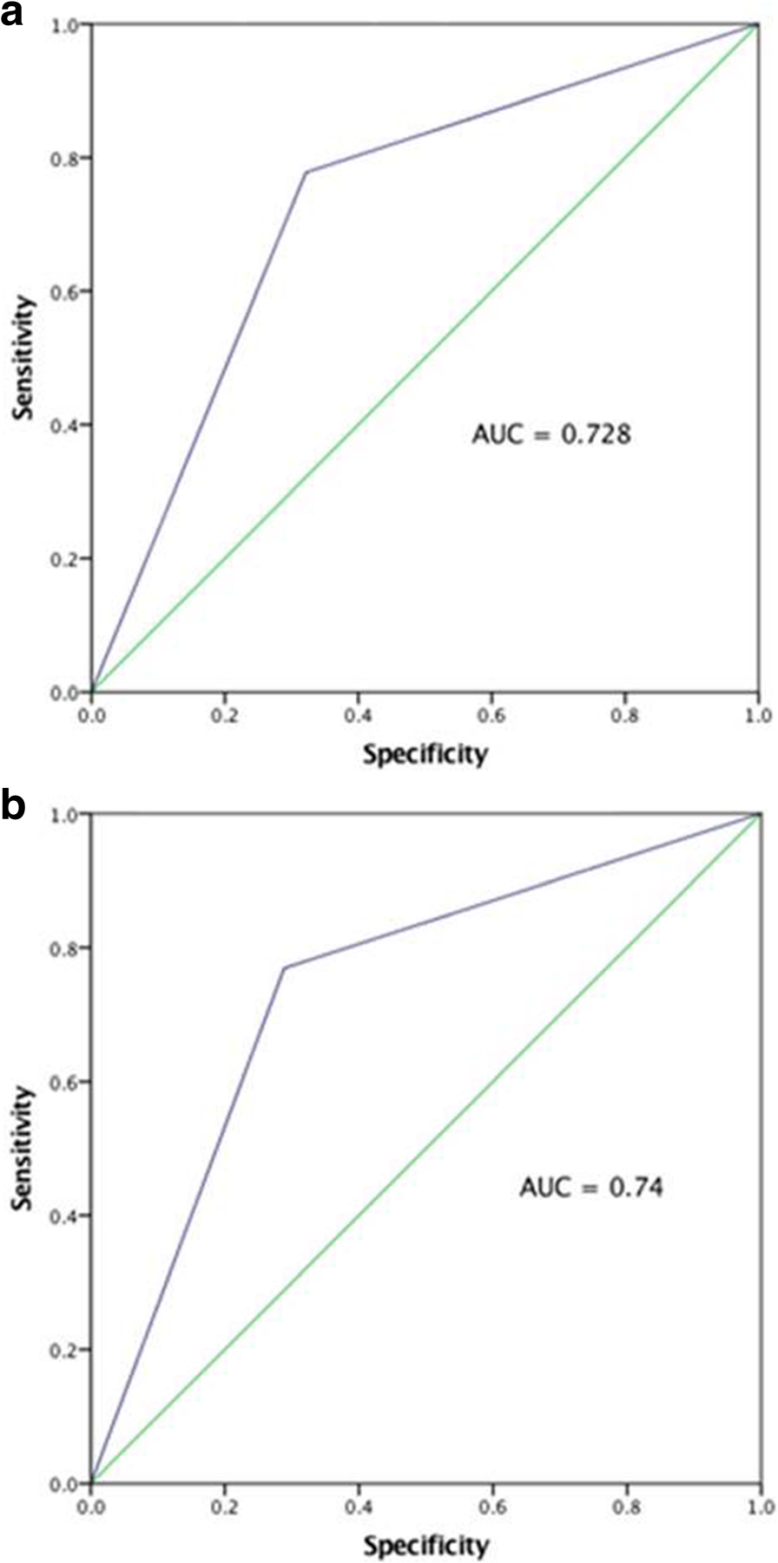
**a** ROC-curve showing relationship between elevated blood lactate (≥ 2.5 mmol/L) and 30-day mortality in patients presenting with heart failure Killip class II-III. **b** ROC-curve showing relationship between elevated blood lactate (≥ 2.5 mmol/L) and 1-year mortality in patients presenting with heart failure Killip class II-III. AUC = area under the curve

In these groups, blood lactate at arrival was associated with both 30-day and 1-year mortality (*p* = 0.021 and *p* = 0.029 respectively) when analysed as a continuous variable. As a dichotomous variable with cut-of ≥2.5 mmol/L, blood lactate was associated with both 30-day (*p* = 0.006) and 1-year (*p* = 0.002) mortality. This association was significant even when excluding patients presenting in Killip class IV (Table 4, Fig. 4).

**Table 4:** Cox Regression Analysis, Killip class II-III

	30-day mortality	1-year mortality
Lactate (per 1 mmol/L)	HR = 1.14 CI = 0.93–1.4	HR = 1.12 CI = 0.94–1.33
Lactate ≥2.5 mmol/L	HR = 5.94 CI = 1.23–28.64*	HR = 6.20 CI = 1.70–22.61*
SBP (per 10 mmHg)	HR = 0.86 CI = 0.67–1.09	HR = 0.89 CI = 0.73–1.08
SBP ≤ 90 mmHg	N.A	N.A
Male sex	HR = 0.61 CI = 0.16–2.28	HR = 0.77 CI = 0.25–2.35
Age (per 5 years)	HR = 1.16 CI = 0.83–1.62	HR = 1.20 CI = 0.91–1.59
BMI (per 5 units)	HR = 0.98 CI = 0.52–1.83	HR = 0.90 CI = 0.52–1.55

*P < 0,05

*HR* hazard ratio, *CI* 95% confidence interval, *BMI* body mass index, *SBP* systolic blood pressure

In patients with Killip class II-III but lactate < 2.5 mmol/L, 30-day mortality was 5% (*N* = 2) and 1-year mortality was 8% (*N* = 3) (Fig. 3). In the comparable group with elevated lactate, mortality was 28% (*N* = 7) at 30 days and 40% (*N* = 10) after 1 year (Fig. 3). This cut-off provided discriminative information on both 30-day (*p* = 0.026 and area under ROC curve 0.73) and 1-year mortality (*p* = 0.002 and area under the ROC curve 0.74).

Systolic blood pressure at presentation was associated with mortality when including all patients with signs of heart failure (*p* = 0.013 and 0.025 at 30-days and 1-year respectively). This relationship was not seen in patients with Killip class II and III.

Regarding hypotension analysed as a dichotomous parameter of systolic blood pressure below, as compared to higher than or equal to 90 mmHg, no association to mortality was found. Neither age, sex nor BMI showed a positive correlation to mortality (*p* > 0.05).

## Discussion

Evaluation of heart failure by Killip classification has been an important adjunct in prognostic evaluation since first described by Killip and Kimball in the early 1960’s [1]. It’s clinical usefulness has been demonstrated several times, most recently by DeGeare et al., in a modern post-revascularization setting in 2001 [3]. The present study extends these observations, and demonstrates that even in patients with AMI presenting with Killip class II and III, lactate levels ≥ 2.5 mmol/L confers a worse prognosis.

In our material, lactate analysed as a continuous variable was significantly related both to short- and long term mortality in patients with acute coronary syndrome and signs of heart failure.

Further, in patients presenting with clinical light to moderate heart failure, defined as Killip class II and III, we found that an increased blood lactate level analysed as a dichotomous parameter with threshold ≥2.5 mmol/L, was associated with increased mortality at both 30 and 365 days even when excluding patients with known negative prognostic factors such as overt cardiogenic shock, hypotension, and out of hospital cardiac arrest, patients seeking care more than 24 h after symptom debut and undergoing rescue PCI.

A control sample of 20 patients classified as Killip class I was analysed, from which 4 patients presented with blood lactate > 2.5 mmol/L. Three of these patient had MI, while one healthy individual underwent a coronary angiography on behalf of suspected unstable angina pectoris with normal result. All where alive after 365 days. Even though several patients classified as Killip I had lactate levels above normal, the mortality of the group as a whole remained as low as 3% after 30 days. This could suggest that an elevated lactate level can be used a predictor of mortality, only when clinical signs of heart failure is present, a question that lies beyond the scope of this study.

It is established that patients presenting with acute coronary syndrome but without overt signs of congestive heart failure (Killip class I) have a relatively favourable outcome (mortality 3% at 30-days in our material, and 2.4% in De Geare and colleagues’ material from 2001) [3]. On the other hand, mortality in cardiogenic shock remains high in spite of historical efforts to improve outcome with means such as inotrope agents, [20] intra-aortic balloon pumps [8], ventricular assist devices [9] and extra corporeal membrane oxygenation (ECMO) [21]. Our data showed a 30-day mortality of 50% in patients with ACS and cardiogenic shock. This level is consistent with comparable international data [5].

Earlier studies on blood lactate have proved its role in risk stratification regarding patients in need of intensive care in general [14, 15] and specifically in patients suffering of sepsis [12, 13, 16]. In patients suffering of acute coronary disease, data have been more inconsistent. Lazzeri and colleagues [18] showed in 2010 that an increased lactate was associated with early mortality in patients presenting with STEMI and advanced Killip class, but could not identify a relationship in patients in Killip class I and II. To the best of our knowledge, though, this study did not separate patients with no signs of heart failure from those with low-grade heart failure, and lactate level was tested as a continuous variable, and not as a dichotomous parameter with a low cut-of point. The same year, Vermeulen et al [22] demonstrated a relationship between blood lactate and short-term mortality in STEMI, but did not exclude patients with advanced Killip class (however, intubated patients were excluded).

Blood pressure analysed as a semi-continuous variable (by steps of 10mHg), was statistically associated with mortality only when including patients classified as Killip class IV. When excluding patients with overt shock or hypotension with signs of peripheral vasoconstriction (Killip class IV), this association was no longer seen. The same result was seen when analysing systolic blood pressure as a dichotomous parameter with threshold 90 mmHg.

The relationship between systolic blood pressure and mortality in the case of acute heart failure has been established previously [5], and lack of significance in this study may have several explanations. Mainly, one could suspect it to be type I error due to few hypotensive patients. Among 77 patients in Killip class II and higher only 6 (8%) were hypotensive at arrival. This number is lower than expected from previous studies, but might be attributed to relatively short transport times and well-developed infrastructure in southern Sweden, and hence relatively short time from symptom debut to arrival at the hospital. Secondly, when analysing blood pressure as a dichotomous parameter, we disregard any differences between moderate and more severe hypotension.

The main limitation of this study is the relatively small number of available subjects from a single centre. The SCAAR registry consists of data manually recorded by the responsible angiography operator, and for a total of 46 (4%) patients were excluded from the study on ground of missing Killip classification. All data were reviewed retrospectively. Data on blood pressure and blood lactate were derived from first recorded values in the patient journal on arrival, but the exact time of measurement or sampling relating to the angiographic procedure cannot accurately be decided retrospectively. In addition, time from symptom debut could possibly affect lactate levels, but is not available in our data. Collecting peak lactate and lactate development could have given additional information. Furthermore, lactate levels are possibly affected by presence of diabetes. Analysing this relationship would have been interesting to analyse further, if not limited by our data number.

Compared with equivalent data from earlier studies [3, 23], the data in our material included relatively few patients presenting with signs of heart failure as a whole, as well as far developed heart failure. Notably, the mortality in patients in Killip class II was higher than those in Killip class III. This difference is probably due to few available data, but could also be a result of imprecise classification. Despite this, the mortality rates in both Killip class IV and Killip classes II/II when assessed together, are in parity or even lower than similar data from previous studies [3, 23].

Patients with acute coronary syndrome and evaluated heart failure in Killip class IV or hypotension have a high mortality rate. In this material, we have identified patients with clinical heart failure without affected hemodynamics, which despite adequate blood pressure have high mortality rates. Rising lactate in these patients could be a marker of tissue hypoxia as a result of peripheral vasoconstriction, as adrenergic compensation still maintains the blood pressure despite an increase in pre-load and falling cardiac output (CO). Could lactate in these cases in fact be a marker of insipient but still compensated cardiogenic shock? Mortality in established cardiogenic shock is still severe. If lactate indeed is an early marker, it might be a useful adjunct in early detection and initiation of treatment to prevent escalation into overt cardiogenic shock.

## Conclusion

Our study suggests that in patients suffering from acute myocardial infarction, a blood lactate level of over or equal to 2.5 mmol/l obtained in the angiographic catheter lab may be used as an adjunct in identifying patients with higher risk of short term death, even in the absence of apparent signs of cardiogenic shock and hypotension. The study was, however, small, and should be regarded as a hypothesis-generating pilot study.

## Abbreviations

AMI: Acute myocardial infarction
BMI: Body mass index
CO: Cardiac output
MI: Myocardial infarction
PCI: Percutaneous coronary interventions
SCAAR: Swedish Coronary Angiography and Angioplasty Registry

## References

[1] Killip T, Kimball JT (1967). Treatment of myocardial infarction in a coronary care unit. A two year experience with 250 patients. Am J Cardiol.

[2] McManus DD, Gore J, Yarzebski J, Spencer F, Lessard D, Goldberg RJ (2011). Recent trends in the incidence, treatment, and outcomes of patients with STEMI and NSTEMI. Am J Med.

[3] DeGeare VS, Boura JA, Grines LL, O'Neill WW, Grines CL (2001). Predictive value of the Killip classification in patients undergoing primary percutaneous coronary intervention for acute myocardial infarction. Am J Cardiol.

[4] Harjola V-P, Lassus J, Sionis A, Køber L, Tarvasmäki T, Spinar J (2015). Clinical picture and risk prediction of short-term mortality in cardiogenic shock. Eur J Heart Fail.

[5] Jeger RV, Radovanovic D, Hunziker PR, Pfisterer ME, Stauffer J-C, Erne P (2008). Ten-year trends in the incidence and treatment of cardiogenic shock. Ann Intern Med.

[6] Lee KL, Woodlief LH, Topol EJ, Weaver WD, Betriu A, Col J (1995). Predictors of 30-day mortality in the era of reperfusion for acute myocardial infarction. Results from an international trial of 41,021 patients. GUSTO-I Investigators. Circ.

[7] Hochman JS, Sleeper LA, Webb JG, Sanborn TA, White HD, Talley JD (1999). Early revascularization in acute myocardial infarction complicated by cardiogenic shock. SHOCK Investigators. Should We Emergently Revascularize Occluded Coronaries for Cardiogenic Shock. N Engl J Med.

[8] Thiele H, Zeymer U, Neumann F-J, Ferenc M, Olbrich H-G, Hausleiter J (2013). Intra-aortic balloon counterpulsation in acute myocardial infarction complicated by cardiogenic shock (IABP-SHOCK II): final 12 month results of a randomised, open-label trial. Lancet (London, England).

[9] Lauten A, Engström AE, Jung C, Empen K, Erne P, Cook S (2013). Percutaneous left-ventricular support with the Impella-2.5-assist device in acute cardiogenic shock: results of the Impella-EUROSHOCK-registry. Circulation Heart Fail.

[10] Goldberg RJ, Samad NA, Yarzebski J, Gurwitz J, Bigelow C, Gore JM (1999). Temporal trends in cardiogenic shock complicating acute myocardial infarction. N Engl J Med.

[11] Mavrić Z, Zaputović L, Zagar D, Matana A, Smokvina D (1991). Usefulness of blood lactate as a predictor of shock development in acute myocardial infarction. Am J Cardiol.

[12] Shapiro NI, Howell MD, Talmor D, Nathanson LA, Lisbon A, Wolfe RE, Weiss JW (2005). Serum lactate as a predictor of mortality in emergency department patients with infection. Ann Emerg Med.

[13] Rimachi R, Bruzzi de Carvahlo F, Orellano-Jimenez C, Cotton F, Vincent JL, De Backer D (2012). Lactate/pyruvate ratio as a marker of tissue hypoxia in circulatory and septic shock. Anaesth Intensive Care.

[14] Jansen TC, van Bommel J, Bakker J (2009). Blood lactate monitoring in critically ill patients: a systematic health technology assessment. Crit Care Med.

[15] Khosravani H, Shahpori R, Stelfox HT, Kirkpatrick AW, Laupland KB (2009). Occurrence and adverse effect on outcome of hyperlactatemia in the critically ill. Critical Care (London, England).

[16] Musikatavorn K, Thepnimitra S, Komindr A, Puttaphaisan P, Rojanasarntikul D (2015). Venous lactate in predicting the need for intensive care unit and mortality among nonelderly sepsis patients with stable hemodynamic. Am J Emerg Med.

[17] Attaná P, Lazzeri C, Chiostri M, Picariello C, Gensini GF, Valente S (2012). Lactate clearance in cardiogenic shock following ST elevation myocardial infarction: A pilot study. Acute Card Care.

[18] Lazzeri C, Valente S, Chiostri M, Picariello C, Gensini GF (2012). Lactate in the acute phase of ST-elevation myocardial infarction treated with mechanical revascularization: a single-center experience. Am J Emerg Med.

[19] Kawase T, Toyofuku M, Higashihara T, Okubo Y, Takahashi L, Kagawa Y (2015). Validation of lactate level as a predictor of early mortality in acute decompensated heart failure patients who entered intensive care unit. J Cardiol.

[20] Belletti A, Castro ML, Silvetti S, Greco T, Biondi-Zoccai G, Pasin L (2015). The Effect of inotropes and vasopressors on mortality: a meta-analysis of randomized clinical trials. Br J Anaesth..

[21] Rihal CS, Naidu SS, Givertz MM, Szeto WY, Burke JA, Kapur NK (2015). 2015 SCAI/ACC/HFSA/STS Clinical Expert Consensus Statement on the Use of Percutaneous Mechanical Circulatory Support Devices in Cardiovascular Care: Endorsed by the American Heart Assocation, the Cardiological Society of India, and Sociedad Latino Americana de Cardiologia Intervencion; Affirmation of Value by the Canadian Association of Interventional Cardiology-Association Canadienne de Cardiologie d'intervention. J Am Coll Cardiol.

[22] Vermeulen RP, Hoekstra M, Nijsten MW, van der Horst IC, van Pelt LJ, Jessurun GA (2010). Clinical correlates of arterial lactate levels in patients with ST-segment elevation myocardial infarction at admission: a descriptive study. Crit Care (London, England).

[23] De Mello BHG, Oliveira GBF, Ramos RF, et al. Validation of the Killip-Kimball Classification and Late Mortality after Acute Myocardial Infarction. Arq Bras Cardiol. 2014;103(2):107–17. doi: 10.5935/abc.20140091. 10.5935/abc.20140091 PMC4150661 25014060

